# Antioxidant Particleboards Produced from Forest By-Products with Application in the Food Packaging Industry

**DOI:** 10.3390/polym17020216

**Published:** 2025-01-16

**Authors:** Raquel A. Fernandes, Nuno Ferreira, Sandro Lopes, Beatriz Freitas, Jorge Santos, Jorge M. Martins, Luisa H. Carvalho

**Affiliations:** 1 ARCP Colab—Rede de Competências em Polímeros, UPTEC—Asprela II, Rua Júlio de Matos, 828/882, 4200-355 Porto, Portugal; nuno.ferreira@arcp.pt (N.F.); sandro.lopes@arcp.pt (S.L.); beatriz.fernandes@arcp.pt (B.F.); jorge.ucha@arcp.pt (J.S.); 2 LEPABE—Faculty of Engineering, University of Porto, Rua Dr. Roberto Frias, s/n, 4200-465 Porto, Portugal; jmmartins@estgv.ipv.pt; 3 ALiCE—Associate Laboratory in Chemical Engineering, Faculty of Engineering, University of Porto, Rua Dr. Roberto Frias, 4200-465 Porto, Portugal; 4 DEMad—Department of Wood Engineering, Polytechnic University of Viseu, Campus Politécnico de Repeses, 3504-510 Viseu, Portugal

**Keywords:** antioxidant, active packaging, polyphenols, particleboards

## Abstract

The food packaging industry is one of the fastest growing sectors of our economy, with a large contribution to environmental concerns due to the extensive use of fossil-derived materials. Combining wood-based materials, such as particleboards, with bio-adhesives may offer a great opportunity to develop sustainable packaging solutions with active antioxidant properties. In the present work, a phenolic extract of poplar bark was produced and bio-adhesives were formulated using citric acid as a cross-linker. The impact of citric acid content on the chemical and bonding properties of bio-adhesives was evaluated. Additionally, the impact of the temperature of curing on their antioxidant capacity was also accessed. The bio-adhesives were applied in the production of particleboards, using poplar veneer particles as raw material. The composite materials exhibit high mechanical resistance, fulfilling the requirement of PB type P1, with remarkable antioxidant activity, opening a possibility to be employed in an active packaging solution.

## 1. Introduction

The food packaging industry is one of the fastest developing sectors, with a 5% annual growth rate in the packaged-food consumption market [1,2]. In 2030, it is expected that this sector will reach 3 trillion €, representing an increase of 100% compared to the market of 2020 [1,2]. One of the major problems associated with this sector is the extended consumption of fossil-derived materials, such as plastic, and glass and metal, resulting in a tremendous environmental impact [1,2]. Therefore, the development of novel and sustainable alternatives has been a point of interest for both scientific and industrial communities, especially focused on wood-based solutions.

Wooden products have been used by the food industry for decades, not only for packaging purposes, but also for transportation and handling [3]. Currently, the wooden packaging solutions that exist in the food industry are based on virgin material, especially in the form of veneers. During the industrial process associated with this transformation of wood into an easily handling material, more than 50% of raw material is wasted (tree bark and the trim-loss fraction of virgin wood), resulting in an inefficient process [4,5]. Consequently, it is required to include this type of residue in an alternative value-chain to improve sustainability and reduce the ecological impact, such as the production of particleboards.

Particleboards (PBs) are a composite material manufactured under heat and pressure from wood and/or other lignocellulosic particles and an adhesive, with relevant application in the furniture and construction industries [6,7]. In recent years, the incorporation of lignocellulosic residues in this material has been extensively studied, proving its feasibility and contributing to changing the paradigm in wood-based industries [6,7]. Additionally, researchers have also been focused on the development of natural adhesives, based on polyphenols (tannins, lignins), proteins, starch, and other polysaccharides, among others [7,8,9,10], to apply in the manufacturing of PBs and to reduce the use of synthetic resins, contributing to the greener character of the final material.

Besides their bonding capacity, polyphenols exhibit other important properties such as antioxidant and antimicrobial activity [11,12,13,14,15], which are an added value concerning food packaging purposes.

One of the most important characteristics of a sustainable packaging system is the capacity to extend a food product’s shelf-life. This property can be achieved by increasing barrier properties of packaging material or by adding chemical agents to prevent perishability, which is designated “active packaging” [16,17,18]. In an active packaging solution, the incorporated compounds allow it to preserve organoleptic or sensory properties of food, usually through an antioxidant and/or an antimicrobial effect [16,17,18]. The use of synthetic compounds with antioxidant effects in food packaging, such as butylated hydroxytoluene (BHT), tert-butyl hydroquinone (TBHQ), and butylated hydroxyanisol (BHA), bring important safety concerns, both at human health and food migration levels [17,19,20]. Therefore, the substitution of these substances by nature-derived alternatives obtained mainly from vegetable and biological sources has been occurring over recent years [17,19,20]. Among all classes of compounds, polyphenols are the most promising alternatives, since they are ubiquitous in nature and their potential as food preservative was already proved [19,20,21].

Up to now, only our research group has performed work on the application of particleboards for food packaging purposes [22,23,24,25]. Different lignocellulosic by-products were evaluated as raw materials to produce bio-based particleboards, namely cardoon stalks and leaves [22], grape canes [23], stalks and vine strains [25], and poplar veneers and bark [24]. In all cases, the physical-mechanical properties were evaluated and promising values were achieved. However, the antioxidant properties of the produced materials have never been considered and, for this reason, this work aims to overcome this limitation. Therefore, this is, to the best of our knowledge, the first report on the antioxidant properties of lignocellulosic-based particleboards for smart packaging solutions for the food industry.

In the present work, particleboards were produced using residues of the wood veneer industry, namely, poplar bark and poplar veneer trims. A phenolic-based adhesive was prepared using poplar bark extract and citric acid. Citric acid (CA) is a dicarboxylic molecule that can act as a hardener in phenolic-based resins, due to cross-linking interactions [23,26,27]. The addition of CA usually leads to enhanced physical-mechanical properties of PBs and also to lower pressing times and temperatures [23,26,27]. Different amounts of citric acid were added (0, 25, 50, 75, and 100 *wt*% in relation to extract dry mass) and the impact on color, pH, viscosity, and bonding properties was evaluated. The bonding capacity of adhesives was accessed through an Automated Bonding Evaluation System (ABES), and the chemistry involved in the curing reaction was assessed though Fourier Transform Infra-Red (FTIR) spectroscopy. The effect of curing temperature (80, 120, 160, and 200 °C) on the chemical and antioxidant properties of the bio-adhesives was also evaluated. The obtained particleboards were also evaluated in terms of physical-mechanical performance and antioxidant capacity.

## 2. Materials and Methods

### 2.1. Raw Materials

Poplar by-products from packaging production (veneers—PV and bark—PB) were provided by FWFi—Freshwood Forms Industry, Lda (Vieira de Leiria, Portugal). The selection of these materials relies on the reduction of wastes generated during the production of traditional poplar veneer packages, in order to improve the sustainability of the overall production process. Both materials were ground at 1500 rpm using a cutting mill (Retsch SN 300, Retsh, Vila Nova de Gaia, Portugal) with a 4 mm square grid and sieved using a vibrating sieve shaker (Retsch AS 200 control, Retsh, Vila Nova de Gaia, Portugal). The fractions of PV and PB particles between 2 mm and 500 µm were oven-dried at 40 °C until reaching the equilibrium moisture. PB particles were used as raw material for the production of the bio-adhesive, while PV particles were applied in the manufacturing of composite panels. Citric acid (≥99.5%, Merck, Rahway, NJ, USA) and sodium hydroxide (≥97.0%, Merck) were used as received.

### 2.2. Production of the Bio-Adhesives

The production of the bio-adhesive was divided into two stages, namely, an alkaline extraction of PB particles and a cross-linking reaction with citric acid. The extraction procedure was carried out at 80 °C in a 10 L borosilicate reactor with mechanical stirring (200 rpm) and a solid/liquid ratio of 1/10 (*w*/*w*). An aqueous solution of sodium hydroxide (1 wt% regarding dry mass of PB particles) was used as an extraction agent. After 1 h of extraction, the mixture was vacuum filtered (100 µm pore size) and the liquid extract was concentrated in a rotary evaporator (Hei-VAP Expert, Heidolph, Schwabach, Germany) until reaching 10 wt% of solid content. The bio-adhesive was obtained by adding different amounts of citric acid (25, 50, 75, and 100 wt% regarding dry mass of extract) to the concentrated extract, maintaining a final solid content of (35.0 ± 0.6 wt%). The bio-adhesives were denoted CA_*X*, where *X* refers to the content of citric acid (0, 25, 50, 75, 100 wt%).

### 2.3. Characterization of the Bio-Adhesives

#### 2.3.1. Viscosity and pH

The viscosity of the bio-adhesives was determined at room temperature, using a Brookfield Digital Viscometer (Model DV-II) equipped with the spindle no 1 at a speed rate of 50 rpm. The measurement was performed three times, and the average viscosity was calculated.

The determination of pH was performed by using a XS instrument pH electrode.

#### 2.3.2. Fourier Transform Infra-Red (FTIR) Spectroscopy

FTIR spectra were recorded on a VERTEX 70 FTIR spectrometer (BRUKER, Billerica, MA, USA) in transmittance mode and equipped with a high sensitivity DLaTGS detector at room temperature. Dried samples were measured in ATR mode with no pre-treatment, using an A225/Q PLATINUM 140 ATR diamond crystal with a single reflection accessory. The spectra were recorded from 4000 to 400 cm^−1^ with a resolution of 4 cm^−1^. All spectra were recorded and processed using OPUS 7.0 software.

#### 2.3.3. Automated Bonding Evaluation System (ABES) Analysis

The bond strength development was evaluated using an ABES (Adhesive Evaluation Systems, Corvallis, OR, USA), according to the standard procedure [28,29]. In order to access the effect of pressing temperature, the bio-adhesives were also subjected to different pressing temperatures (60, 80, 120, 140, 160, 180, 200 °C) for 120 s. Previously conditioned (one week at 20 °C and 53% relative humidity) wood veneer samples (*Fagus sylvatica* L., with 0.7 mm of thickness, standard wood sample for ABES measurement) were cut into 117 mm × 20 mm strips using a pneumatically driven sample cutting device.

For the test, two probes were glued along the fiber direction using 10 mg of bio-adhesive with a 100 m^2^ overlap region. After hot-pressing, the probes were pulled, and the maximum shear strength was determined. Measurements were conducted in triplicate for each pressing time and the results were averaged.

#### 2.3.4. Antioxidant Activity

The antioxidant activity of the extracts was determined by means of the FRAP (ferric reducing/antioxidant power) assay, according to Szöllösi and Szöllösi-Varga [30]. The relative antioxidant activity of the BLE extract was calculated from the calibration curve of L-ascorbic acid (0.1; 0.2; 0.3; 0.4; 0.5; 0.6 mmol/l), and the result was expressed as nmol ascorbic acid equivalent (AAE) per mg of BLE extract (on a dried basis). The analyses were conducted in triplicate and the mean value calculated.

#### 2.3.5. Degree of Cure

In order to assess the impact of temperature on the curing process, CA_*X* adhesives were oven-dried at different temperatures (80, 120, 160, and 200 °C) to promote their curing. The “cured” solid resins were then redissolved in distilled water and filtered to assess the insoluble fraction (cured resin) through Equation (1):

(1) $$\mathrm{Insoluble}\;\mathrm{fraction}\;\left(\%\right)=\frac{\mathrm{Solid}\;\mathrm{residue}\;\left(g\right)}{\mathrm{Initial}\;\mathrm{mass}\;\left(g\right)}\times 100$$

The analyses were conducted in triplicate and the mean value calculated.

### 2.4. Particleboard Manufacturing and Characterization

CA_*X* adhesives (35.0 ± 0.6% of solid content) were mechanically blended with PW particles, with a resin load of 10% (*w*/*w* on a dry mass basis). One layer of particleboard mat was hand-formed in an aluminum deformable container of 250 × 250 mm^2^, maintaining a regular spread over the entire area. Therefore, the particleboards were pressed in a batch hot-press at 160 °C for 10 min, with a target density of 700 kg m^−3^ and fixed thickness (4 and 8 mm), using metallic blocks as stoppers. The panels were denoted PBCA_*X*, depending on the bio-adhesive tested.

#### 2.4.1. Physical-Mechanical Performance

The physical-mechanical properties of obtained composite panels were evaluated according to European standards in terms of density (NP EN 323:2002 [31]), moisture content (NP EN 322:2002 [32]), internal bond strength (IB; NP EN 319:2002 [33]), bending strength and modulus of elasticity (BS and MOE, respectively; NP EN 310:2002 [34]), and thickness swelling (TS; NP EN 317:2002 [35]). Composite panels were classified according to the standard NP EN 312:2010 [36]. All the determinations were performed in triplicate and the results were averaged.

#### 2.4.2. Antioxidant Activity

The antioxidant activity of particleboards was quantified through the free radical scavenging activity of 1,1-diphenyl-2- picrylhydrazyl (DPPH•) [37]. In this measurement, 3 mL of the DPPH• methanolic solution (0.60 mM) was mixed with 0.250 g of particleboard and left in the dark at room temperature for 30 min. After the reaction, the absorbance was measured at 517 nm using a UV-vis Peak Instruments T-9100 spectrophotometer. The antioxidant activity (AA) was calculated according to Equation (2):

(2) $$\mathrm{AA}\;\left(\%\right)=\frac{A_{s}-A_{b}}{A_{b}}\times 100,$$

where A_b_ is the absorbance of the blank (distilled water) and A_s_ is the absorbance of the sample. The analyses were conducted in triplicate, and the mean value was calculated.

### 2.5. Statistical Analysis

All determinations were conducted in triplicate, and the data are presented as the means ± standard deviations. The effect of citric acid on the physical-mechanical properties of particleboards (density, MC, IB, BS, MOE, and TS) and the effect of the temperature of curing on the FTIR peak areas, the degree of curing, and antioxidant activity were statically evaluated (*p*-value < 0.05) by one-way analysis of variance (ANOVA) using XLStat software and Tukey’s test for differences of means. Similarly, the impact of pressing temperature on the same parameters and antioxidant activity was also statistically evaluated through one-way ANOVA and Tukey’s test. Regarding the impact of thickness on the antioxidant activity and citric acid content of panels, statistical analysis was performed by two-way analysis of variance (ANOVA) at *p*-value < 0.05 using XLStat 2024.4.0 software.

## 3. Results and Discussion

### 3.1. Characterization of the Bio-Adhesives

**Figure 1:**
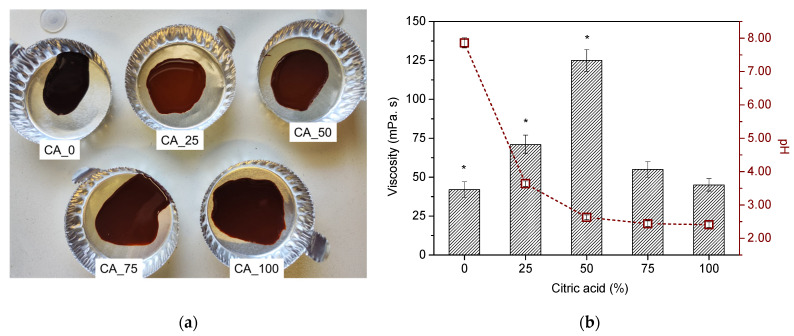
(**a**) Photograph of CA_*X* adhesives (X = 0, 25, 50, 75, and 100). (**b**) Effect of citric acid amount (0, 25, 50, 75, and 100%) on viscosity and pH. Samples subscripted by * are significantly different in terms of viscosity and pH at *p* < 0.05.

Poplar bark extract obtained through alkaline hydrolysis was mixed with different ratios of citric acid (25, 50, 75, and 100 wt% regarding dry mass of extract) to obtain a sustainable bio-adhesive for particleboard manufacturing. The addition of citric acid not only changed the color of the bio-adhesive, turning it lighter (Figure 1a), but also affected its rheological properties, such as viscosity and pH. Figure 1b presents the effect of the citric acid amount on these two parameters.

As can be observed, poplar bark extract (CA_0; Figure 1b) presents a slightly alkaline pH (7.86, Figure 1b) due to the use of aqueous NaOH as extraction agent. With the addition of citric acid, pH of the bio-adhesive suffered a drastic decrease of up to 50% of CA to ca. 2.63 (CA_50; Figure 1b). With 75% and 100% of CA, no significant variation was observed in terms of pH (Figure 1b).

Regarding viscosity, a great impact of CA content was also observed, with a sharp increase (*p* < 0.05) from 42 to 125 mPa s (CA_0 and CA_50, respectively). Above 50%, the addition of CA promoted a significant decrease in viscosity to c.a. 45 mPa s (CA_100; Figure 1b).

According to the literature, the addition of carboxylic acids (as citric acid) to phenolic-rich solutions promotes polymerization mechanisms through esterification [38,39,40,41]. This chemical phenomenon increases the molecular weight of compounds, promoting the formation of a cross-linked network and, consequently, increasing viscosity. When the cross-linking agent is in excess, the ratio of hydroxyl:carboxyl groups significantly decreases, limiting the polymerization process and consequently, lowering viscosity.

**Figure 2:**
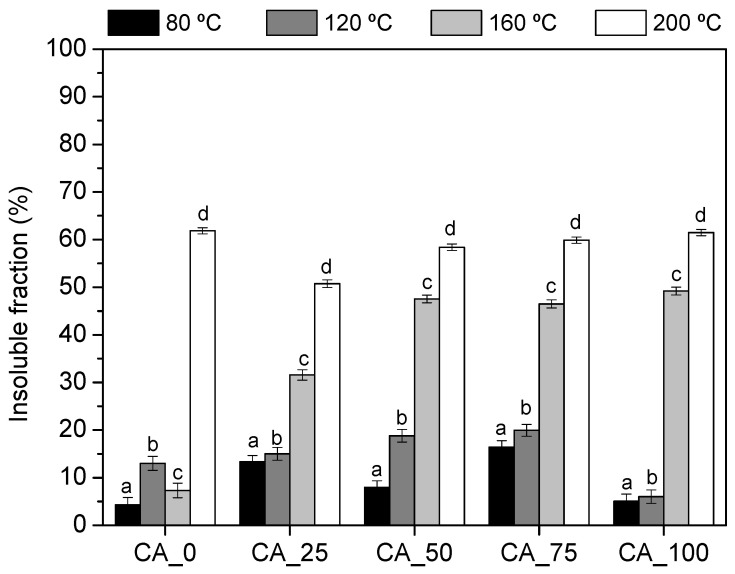
Solubility in water of the CA_*X* adhesives (*X* = 0, 25, 50, 75, and 100). Samples subscripted by different letters are significantly different in terms of temperature at *p* < 0.05.

The curing behavior of CA_X adhesives was evaluated by oven-drying them at different temperatures (80, 120, 160, and 200 °C) to promote their curing. After this, the cured resins were redissolved in water in order to access the impact of the citric acid amount on the efficiency of the curing process. When a resin is completely cured, its components are not able to dissolve into water. Contrariwise, if an adhesive is not totally cured at a specific temperature, the uncured fraction will be solubilized in water. Figure 2 presents the results obtained on the determination of the insoluble fraction of each adhesive.

The solubility of the “cured” bio-adhesives is related to their hydrophobicity and to the degree and type of cross-linking achieved. According to Figure 2, it is possible to confirm the influence of citric acid as a cross-linking agent and the relevance of the curing temperature on the kinetics of the reaction. Without citric acid (CA_0), polyphenolic extract presents an insoluble fraction of 7% after curing up to 160 °C (Figure 2). By increasing the curing temperature to 200 °C, the amount of insoluble matter significantly (*p* < 0.05) increased, reaching c.a. 62% (Figure 2), due to the occurrence of self-condensation reactions of polyphenols [9,23,24].

With the addition of citric acid, a clear impact was noticed on the temperature of curing. Up to 120 °C, the behavior of CA_25, CA_50, and CA_75 adhesives was quite similar, reaching a 15–25% insoluble fraction after curing (Figure 2). In the case of CA_100, the solubility was slightly higher up to 120 °C (Figure 2), which may indicate a lower degree of reaction between citric acid and polyphenols. Increasing temperature to 160 °C, the bio-adhesives containing citric acid exhibit a sharp increase in their insoluble fraction, from 8% (CA_0, Figure 2) to 30% (CA_25, Figure 2) and 50% (CA_50, CA_75, and CA_100, Figure 2). This finding showed that citric acid acts as a cross-linking agent by reducing the temperature required to obtain a high degree of cross-linking and, consequently, a high water-insoluble fraction at low curing temperatures (120–160 °C).

**Figure 3:**
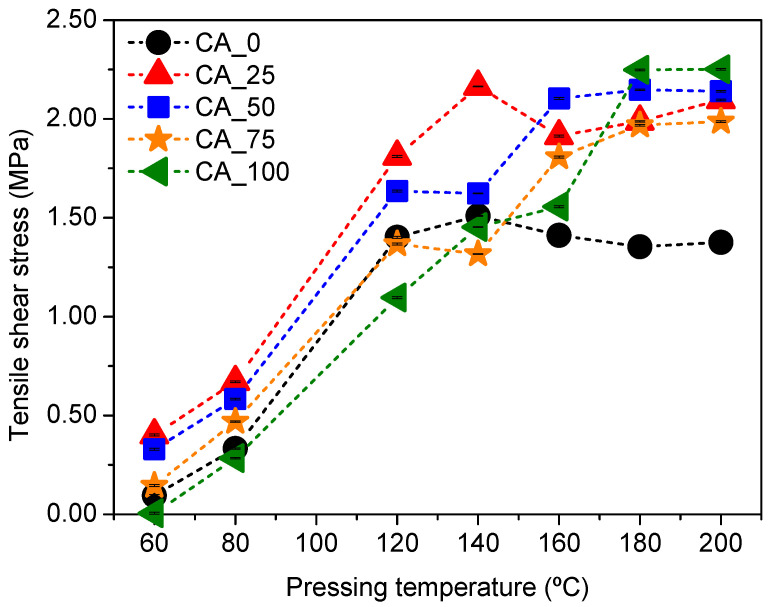
Effect of pressing temperature on shear strength of CA_*X* bio-adhesives (X = 0, 25, 50, 75, and 100).

To confirm the effect of citric acid on the reactivity of polyphenolic extract, the bonding capacity of CA_X adhesives was evaluated using an ABES device, through the simulation of a hot-pressing process at defined conditions of temperature and pressure. The tensile shear strength of all bio-adhesives (35.0 ± 0.6 wt% of solid content) was tested at different pressing temperatures (from 60 °C to 200 °C) by fixing 120 s of pressing time in all cases. Figure 3 presents the obtained results.

As can be observed, pressing temperature has a direct impact on the bonding performance of CA_X adhesives (Figure 3). In the absence of citric acid (CA_0), the maximum shear strength was c.a. 1.50 MPa by pressing at 140 °C (Figure 3). With the addition of 25 wt% of citric acid (CA_25), the maximum shear strength achieved increased to c.a. 2.20 MPa at the same temperature (Figure 3). In both cases, a plateau in maximum shear strength is reached up to 140 °C, with no significant variation up to 200 °C (Figure 3). However, a higher amount of citric acid (50 and 75 wt%) promoted a clear change in the profile of curing. According to the results, these bio-adhesives exhibited a two-step mechanism of bonding, with two different plateaus: the first at 120–140 °C and a second one at 160–200 °C (Figure 3). This finding is related with the cross-linking reactions that occur between carboxyl groups of citric acid and hydroxyl groups of both polyphenols and lignocelulose components [22,23,41,42,43,44]. Several authors have concluded that to produce a composite material with superior physical-mechanical performance using a citric-acid-containing adhesive, it is necessary to adopt hot-pressing temperatures near 180–200 °C and pressing times of 10–15 min to guarantee the polymerization reaction [45,46]. By increasing citric acid content, the quantity of carboxylic groups available to promote this reaction is higher and, consequently, their contribution to the overall bonding effect of the bio-adhesive is enhanced. However, the maximum shear strength achieved by CA_75 (1.96 MPa; Figure 3) was lower than that of CA_50 (2.14 MPa; Figure 3), which may be attributed to the existence of free citric acid molecules in this adhesive. The excess of citric acid favors its direct interaction with hydroxyl groups of wood and hinders cross-linking reactions, decreasing the resistance of the wood/adhesive joint. In fact, the results observed using CA_100 also corroborate this statement; the free citric acid molecules are more prevalent in this case and, consequently, the wood/bio-adhesive interactions are mostly replaced by citric acid/wood interactions, which may also explain the unique plateau at 180 –200 °C.

**Figure 4:**
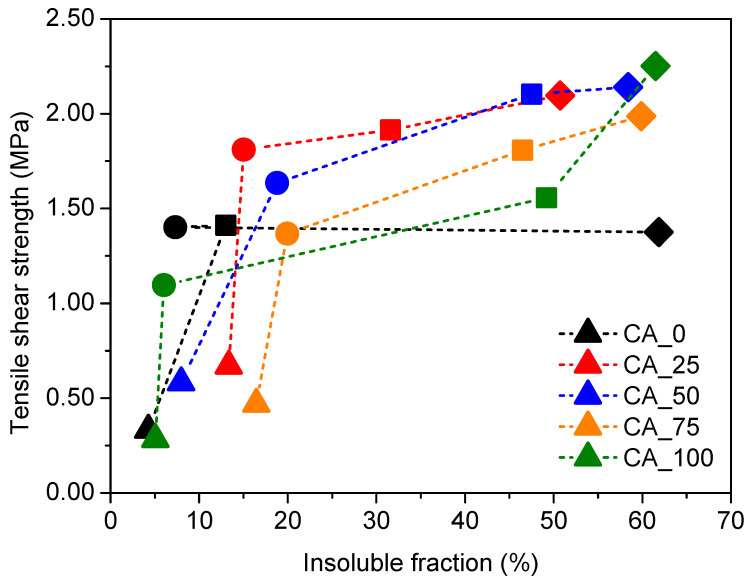
Relation between the insoluble fraction of CA_*X* adhesives (*X* = 0, 25, 50, 75, and 100) and shear strength at a specific temperature of curing, namely 80 °C (triangle), 120 °C (circle), 160 °C (square), and 200 °C (diamond).

A direct relation was established between the amount of insoluble fraction and the shear strength obtained through ABES at a specific temperature of curing (80, 120, 160, and 200 °C), and the results are shown in Figure 4.

As can be observed, all CA_X adhesives follow the same tendency, with an increase in tensile strength associated with an increase in insoluble fraction (Figure 4). Additionally, it was also noticed that adding citric acid up to 50% resulted in an improvement in adhesive curing (higher shear strength and insoluble fraction; Figure 4). With over 50% of citric acid, the bonding efficiency is decreased, with lower insoluble fraction/tensile shear strength ratios (Figure 4).

**Figure 5:**
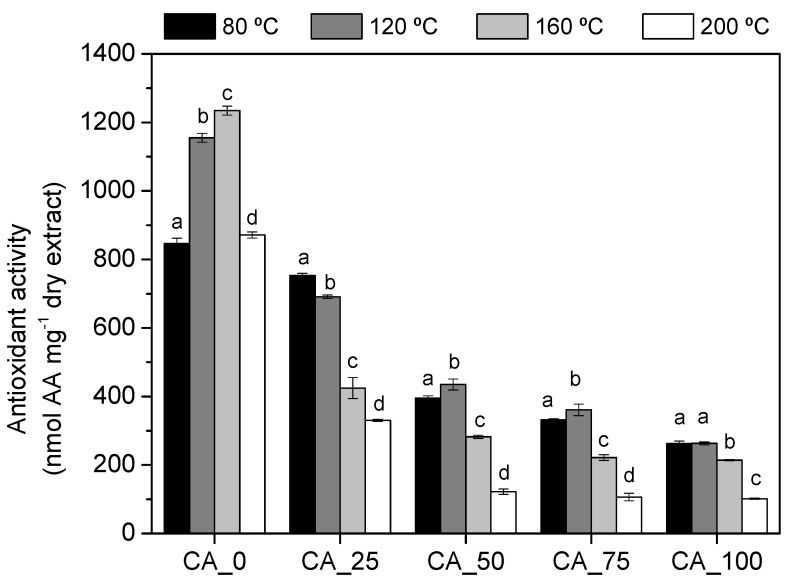
Antioxidant activity measured by FRAP of the water-soluble fraction of the CA_*X* adhesives (*X* = 0, 25, 50, 75, and 100). Samples subscripted by different letters are significantly different in terms of temperature at *p* < 0.05.

The next step of the study was to evaluate the impact of temperature of curing on the antioxidant activity of the bio-adhesives. In a previous work performed by our research group [24], poplar bark extracts were shown to have interesting antioxidant activity. Taking this into account, the present study is focused on the evaluation of the potential antioxidant activity of the bio-adhesive formulated with poplar bark extract and citric acid. For this purpose, the bio-adhesives were cured at different temperatures (80, 120, 160, and 200 °C) and the antioxidant activity of the water-soluble fraction was evaluated using FRAP methodology. The results are shown in Figure 5.

According to the results, poplar bark extracts (CA_0) showed high thermal stability in terms of antioxidant activity, with antioxidant activity increasing even when the extract solution was dried at 120 and 160 °C (Figure 5). This fact may indicate that the extract has compounds with higher antioxidant activity that do not react by self-condensation at these temperatures, maintaining their properties. Regarding the effect of citric acid, the antioxidant activity of the soluble fraction of all bio-adhesives decreased with the increasing percentage of citric acid used in the formulation (Figure 5). In general, when citric acid was used in the bio-adhesive formulation, the curing temperature is lowered, which has a negative effect on the antioxidant activity of the soluble compounds present in the cured resins. This finding may indicate that polyphenols are mainly responsible for the antioxidant activity of the bio-adhesives and, after the polymerization reaction with citric acid, these molecules lost their antioxidant capacity.

**Figure 6:**
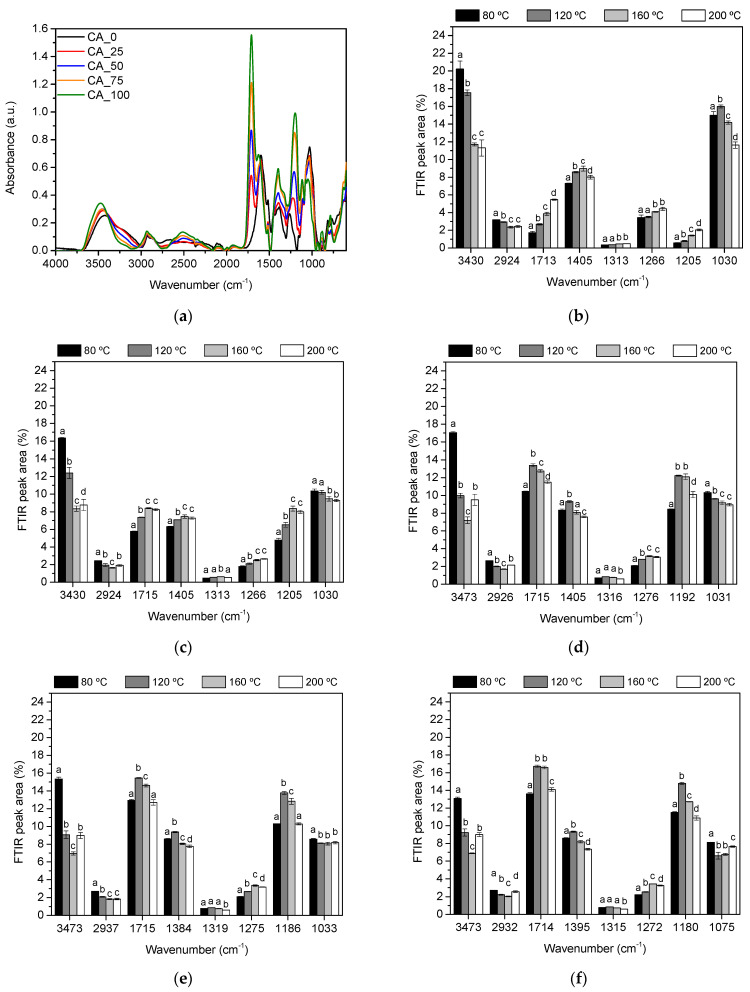
(**a**) FTIR spectra of CA_*X* bio-adhesives (X = 0, 25, 50, 75 and 100). FTIR peak area (%) of characteristic bands of (**b**) CA_0, (**c**) CA_25, (**d**) CA_50, (**e**) CA_75, and (**f**) CA_100. Bars subscripted by different letters are significantly different (*p* < 0.05) in terms of temperature.

FTIR-ATR analyses were performed in order to investigate the polymerization reaction between phenolic compounds and citric acid. In this way, poplar bark extract (CA_0) with 35.0 ± 0.6 wt% of solid content was mixed with 25 wt%, 50 wt%, 75 wt%, and 100 wt% of citric acid (regarding dry mass of extract) and the resulting solutions were oven-dried at 60 °C for 24 h. The absorption band associated with the vibration of the aromatic polyphenol ring (at 1600 cm^−1^) was considered for normalization of all FTIR spectra. Figure 6a shows the obtained spectra.

The FTIR spectra of all bio-adhesives (Figure 6a) exhibit the characteristic bands reported in previous work by the research group, confirming the presence of hydrolysable tannins and sugars as the main compounds, with condensed tannins and proteins present in smaller proportions [44].

The effect of temperature of curing on the chemical properties of CA_*X* adhesives was exhaustively studied through FITR. For this purpose, the effect of the curing temperature on the relative areas of the main bands involved in the curing reaction of the bio-adhesives was evaluated (Figure 6b–f).

The target bands were selected on the basis of previous studies carried out by this research group [22,23,24]: the band at 1713–1715 cm^−1^ due to the carbonyl groups (C=O) in the carboxylic acid groups; the band at 3430–3473 cm^−1^ due to the OH stretching vibrations; the band at 2924–2937 cm^−1^ associated with the stretching vibration modes of the CH_2_ groups; the band at 1384–1405 cm^−1^ due to the deformation vibration of the carbon-carbon bonds in the phenolic groups; the band at 1313–1319 cm^−1^ due to the Ph–CHR–OH deformation; the band at 1266–1275 cm^−1^ due to the C–O vibration; the band at 1180–1205 cm^−1^ associated with the C–O stretching of polyphenols; and the band at 1030–1075 cm^−1^ associated with the C–O vibrations mainly of sugars.

In the study of the self-condensation reaction of poplar bark extract (Figure 6b), it was observed that the relative area of the bands due to the OH stretching vibrations (at 3430 cm^−1^) decreased with temperature, due to the participation of the hydroxyl and methylol groups of the polyphenol in the condensation reaction [47,48]. On the other hand, it was also observed that the band due to the characteristic carbonyl groups (C=O) (1713 cm^−1^) of the hydrolysable tannins [49] increased with temperature, indicating that the polymerization process of this type of tannin involves the formation of methylene ester bonds. This fact was confirmed by the observed changes in the relative area of the bands at 1205 cm^−1^ and 1266 cm^−1^, associated with the vibration of the CO bonds of the –O– (C=O) groups, which followed the same trend with temperature. It can also be observed that temperature has an effect on the bands around 1030 cm^−1^, associated with the C–O vibrations mainly of the sugars, which initially increase with temperature due to the participation of the sugars in the self-condensation process, but then decrease due to thermal degradation processes.

For the bio-adhesives formulated with citric acid (Figure 6c–f), the main differences due to the effect of temperature were observed in the OH band (3430–3473 cm^−1^); as above, citric acid shifted the OH band to higher wavenumber values and increased the relative area band reduction at 120 °C and 160 °C, due to the enhanced curing reaction at lower temperatures. However, percentages of citric acid above 50 wt% did not enhance the reduction in the OH band, indicating that citric acid was not involved in the cross-linking reaction of the polyphenols over 50 wt%.

Regarding the formation of methylene ester bonds (1714–1715 cm^−1^ and 1180–1205 cm^−1^), in all adhesives formulated with citric acid (Figure 6b–f) the relative area of the bands characteristic of this type of bond was greater than in the adhesive formulated with poplar extract alone. Regarding the effect of temperature, the increase above 120 °C has an effect only on the bio-adhesive CA_25 (Figure 6c), but in the adhesives formulated with higher percentages of citric acid (50–100 wt%; Figure 6d–f) the area of the bands increases only in the temperature range of 80–120 °C and remains the same or decreases at higher curing temperatures.

This research work confirmed that citric acid is a good cross-linking agent for poplar bark extracts by reducing the temperature required for the curing reaction (as previously observed through ABES; Figure 4) and that increasing the percentage of citric acid above 50 wt% did not improve the curing reaction in terms of the degree of cross-linking achieved or the reaction temperature.

### 3.2. Physical-Mechanical Evaluation of Particleboards

**Figure 7:**
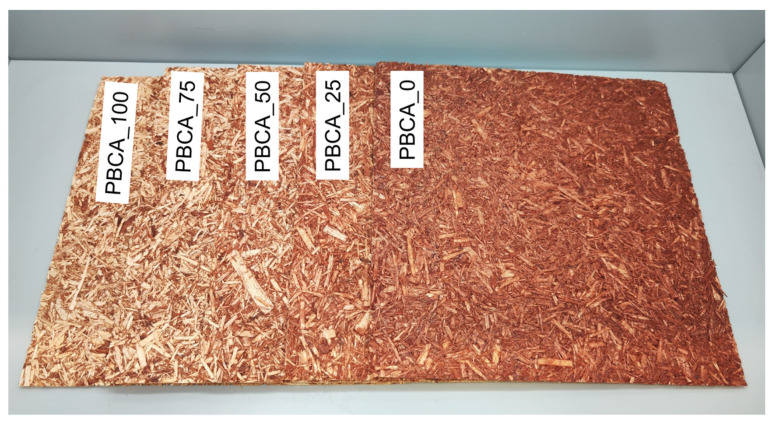
Photograph of the composite panels produced using the CA_*X* bio-adhesives (*X* = 0, 25, 50, 75 and 100).

The bio-adhesives produced were tested in the manufacturing of particleboards, using poplar particles as raw material. The composite panels were hot-pressed at 160 °C for 10 min at a fixed thickness of 4 mm. Figure 7 presents the produced composite panels, using CA_0, CA_25, CA_50, CA_75, and CA_100 adhesives.

As can be observed, increasing CA content in the panel leads to a lighter color of the obtained material, which is explained by the color variation observed in the CA_*X* bio-adhesives (Figure 1a). After conditioning, the panels were tested in order to access their physical-mechanical properties, namely, density, moisture content (MC), internal bond strength (IB), bending strength (BS), and modulus of elasticity (MOE). Table 1 presents the obtained results for all composite materials.

As expected, all composite panels present similar (*p* < 0.05) density, ca. 700 kg m^−3^ (Table 1). In terms of MC, the statistical analysis indicates a significant (*p* < 0.05) difference between materials, with an increase in MC from 4.7% (PBCA_0, Table 1) to 5.4% (PBCA_100; Table 1) with the increase in the citric acid content of CA_*X* adhesive (from 0 to 100 wt%) being observed. This fact may be related to the hygroscopic character of tricarboxylic acids, such as citric acid, that easily absorb water from air, consequently leading to higher equilibrium moisture of composite materials [50].

Regarding mechanical resistance, PBCA_0 achieved an IB of 0.25 MPa (Table 1). With the increase in citric acid content to 25 and 50 wt% (PBCA_25 and PBCA_50, respectively), IB suffers a significant (*p* < 0.05) increase to 0.31 and 0.41 MPa, respectively (Table 1). As previously observed through FTIR analysis (Figure 6), ester bonds between polyphenols and citric acid favor cross-linking interactions of wood and the bio-adhesive, increasing the resistance of particleboards [41]. However, increasing citric acid content to 75 and 100 wt% (PBCA_75 and PBCA_100) leads to a sharp decrease in IB, from 0.41 MPa to 0.32 and 0.33 MPa, respectively (Table 1), which is related to the excess citric acid. At over 50 wt% of citric acid, the bio-adhesive has a great number of free citric acid molecules that are not bonded with polyphenols, as previously confirmed by FTIR analysis (Figure 3). Therefore, the highly hydrophilic character of citric acid may favor its interaction with the hydroxyl groups of PV particles, limiting the chemical bonding with the polymerized polyphenols. This fact may lead to a decrease in the efficiency of wood/bio-adhesive interactions and, consequently, to a reduced mechanical resistance.

In the case of bending properties, the addition of citric acid leads to an improvement in particleboard performance in terms of bending strength and modulus of elasticity (BS and MOE, respectively) up to 75 wt% (Table 1). This finding may indicate that free citric acid may have a positive impact over flexural properties of the composite material, maintaining moisture and reducing stiffness. The same tendency is observed in terms of water resistance, with a sharp decrease in thickness swelling, from total disintegration to 85% with the addition of 75 wt% of citric acid (Table 1). The physical-mechanical performance of citric acid-based particleboards fulfills the requirements of EN 312:2010 for type P1 particleboards (3 mm < thickness < 6 mm) in terms of IB (Table 1), with interesting values of BS (Table 1).

The bonding performance of CA_X adhesives was also evaluated in the manufacturing of particleboards with higher thickness. For this, a set of composite materials with 8 mm thickness was produced using 10 wt% of adhesive (dry basis, regarding dry mass of panel) and maintaining target density (700 kg m^−3^). The physical-mechanical properties of the panels were evaluated and the results are shown in Table 2.

All panels presented a similar density (*p* < 0.05), with a slight increase in MC for citric acid content ≥ 50 wt% (Table 2), as previously observed for particleboards with a 4 mm thickness (Table 1). As expected [51], by increasing thickness to 8 mm, IB drastically reduced (Table 2). However, the impact of citric acid content on the performance of the bio-adhesives followed the same tendency as observed for 4 mm particleboards, with a significant (*p* < 0.05) increase from 0.07 MPa to 0.19 MPa with 50 wt% of citric acid (Table 2). Regarding flexural properties, the results observed for BS (Table 2) and MOE (Table 2) also corroborate this finding, with a significant (*p* < 0.05) impact of citric acid content over 50 wt%. Additionally, increasing particleboards’ thickness allowed a more elastic composite material to be produced, with improved capacity for being molded.

The use of polyphenolic-based adhesives in the manufacturing of PBs presents a common drawback associated with water resistance [52]. Nevertheless, the use of hardeners or cross-linking agents, such as citric acid, may improve the performance of the composite material when it is subjected to high-humidity conditions [40,42]. According to Table 2, PBCA_0 suffered total disintegration after being immersed in water, confirming the weak resistance of polyphenolic-based adhesive to water. By adding citric acid up to 50 wt%, the water resistance of PBCA_25 and PBCA_50 significantly (*p* < 0.05) increased, resulting in lower TS values after 1 h (Table 1). However, for 75 and 100 wt% of citric acid, no impact was noted in terms of water resistance, maintaining TS values over 130% after 1 h (Table 1), which is considered a limiting property regarding the potential industrial application of these composite materials in the packaging industry.

According to the literature [45,46,53,54], the physical-mechanical properties of PBs depend not only on their composition, but also on the conditions adopted during the manufacturing process, such as time and temperature of pressing. In fact, pressing temperature usually allows an improved performance of this type of material regarding water resistance to be achieved, once the chemical structure of wood components is modified [55,56]. At equilibrium conditions, wood adsorbs humidity from air, creating hydrogen bonds with lignin, cellulose, and hemicellulose [55,56]. When wood is subjected to heat treatment, OH groups available to establish these hydrogen bonds decrease due to the degradation of hemicellulose and lignin, and hydrophobic oxygen-acetyl groups are formed between wood fibers, limiting the swelling of the material [55,56].

Therefore, the effect of pressing temperature (180, 200, and 220 °C) was assessed in the manufacturing of PBCA_50 with 8 mm, by fixing pressing time at 10 min in order to guarantee the total cure of the bio-adhesive. Table 3 presents the obtained results of the produced materials.

As expected, all the produced panels presented similar (*p* < 0.05) density (c.a. 700 kg m^−3^; Table 3) and no difference was also noted in terms of moisture content (c. a. 4.8%, Table 3). However, thickness swelling was significantly (*p* < 0.05) improved by increasing pressing temperature (Table 3). In fact, a sharp reduction was observed in the TS value from 142% to 38% by increasing pressing temperature from 160 °C to 200 °C (Table 3), which is in accordance with previous reports for wood-based materials [45,46,51,57]. At 220 °C, the impact of pressing temperature on TS was negligible (*p* < 0.05) when compared to the results achieved using 200 °C (Table 3). In terms of mechanical properties, no significant (*p* < 0.05) impact was observed on IB, BS, and MOE values by increasing pressing temperature from 160 °C to 200 °C (Table 3). Nevertheless, at 220 °C, the mechanical properties were substantially improved (*p* < 0.05), fulfilling the requirements of PB type P1 (PB for general use under dry conditions) according to EN 312:2010.

### 3.3. Antioxidant Properties of Particleboards

**Figure 8:**
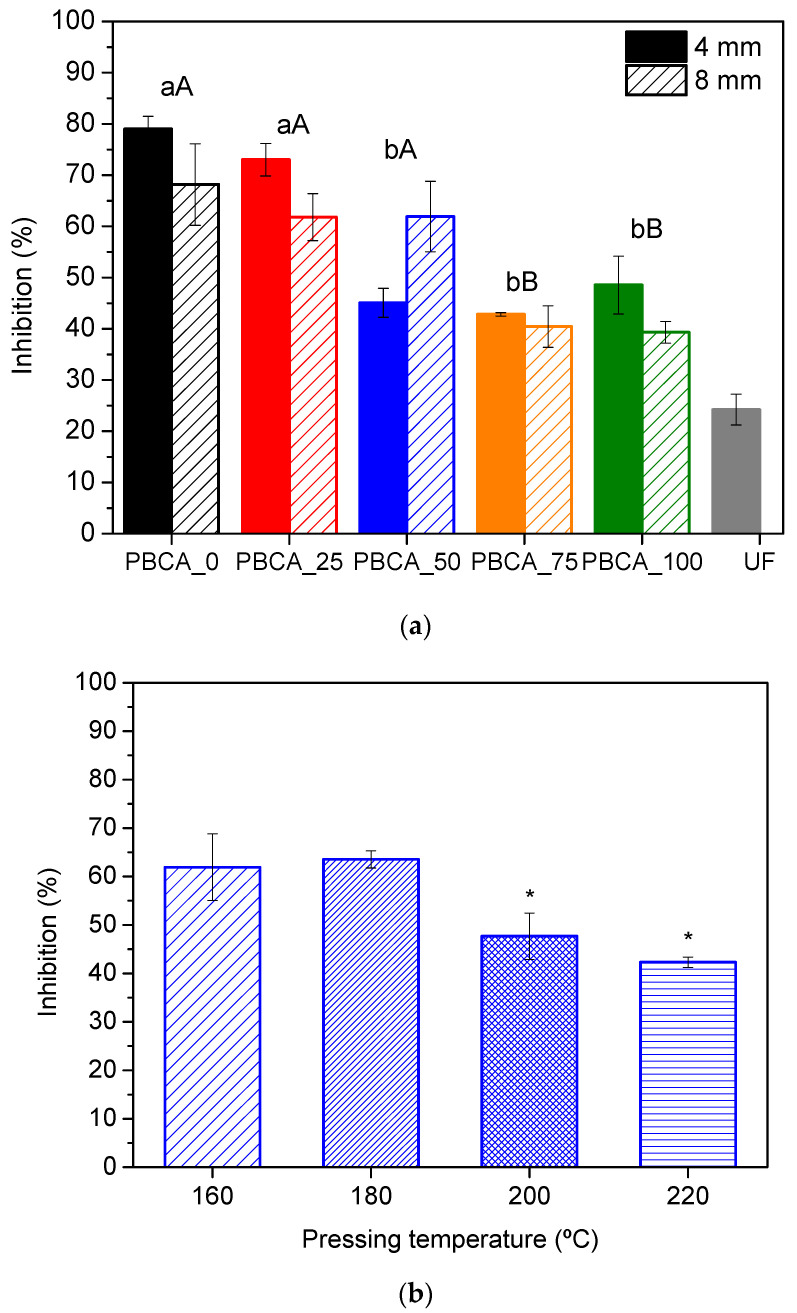
(**a**) Effect of thickness and citric acid content on the antioxidant activity of PBCA_X composite panels and standard UF particleboard. Different letters above columns refer to significantly different values at *p* < 0.05 depending on the thickness (capital letters—8 mm and lowercase letters—4 mm particleboards). (**b**) Impact of pressing temperature on the antioxidant activity of PBCA_50 composite panel. * refers to significantly different columns at *p* < 0.05.

All composite panels were subjected to the determination of antioxidant activity using scavenging DPPH**•**. The impact of citric acid content was evaluated, as well as the thickness and pressing temperature of particleboards. A standard particleboard was produced using a urea-formaldehyde (UF) commercial resin for comparison purposes. Figure 8 presents the results in terms of inhibition of oxidation.

According to Figure 8a, PBCA_*X* panels presented enhanced antioxidant activity (≥ 40% of inhibition, Figure 8a) when compared to standard composite material containing UF resin (c.a. 24% of inhibition, Figure 8a), revealing its potential as a packaging material. This fact is related with the polyphenolic nature of the bio-adhesives, which act as sacrificial agents, preventing/limiting the oxidation of other chemical species. The thickness of the panels did not induce a significant (*p* < 0.05) variation in the antioxidant activity, reaching similar values by using the same bio-adhesive (Figure 8a). In the case of PBCA_0 and PBCA_25, no significant impact was noticed on the antioxidant capacity of the materials (Figure 8a). However, after increasing citric acid content in the bio-adhesive over 50 wt%, a sharp reduction was observed in the inhibition efficiency (Figure 8a). This variation corroborates the central contribution of polyphenols to limiting oxidation, with a negligible impact of free citric acid. As previously confirmed through FTIR analyses (Figure 6), for up to 25 wt% of citric acid, an excess of polyphenols occurred in the bio-adhesives regarding citric acid molecules, leading to a predominance of unreacted polyphenols available for oxidation. By increasing citric acid content to ≥50 wt%, the fraction of unreacted polyphenols decreased, which means that few hydroxyl groups are available to be oxidized and, consequently, lower antioxidant activity is observed. These findings not only confirm the undeniable relation between polyphenols and antioxidant properties, but also prove the negligible contribution of citric acid, which enhances the advantages of using forestry extracts as a binding agent to produce composite materials for food packaging.

## 4. Conclusions

Packaging production by-products from wood peeling (poplar bark and poplar veneers) were successfully applied in the manufacturing of antioxidant composite panels for the food packaging industry. Poplar bark was used as the raw material for bio-adhesive production, by combining it with citric acid, a natural cross-linker. The impact of citric acid content on chemical and bonding properties of the bio-adhesives was evaluated through viscosity measurements, FTIR analysis, and ABES. It was found that citric acid content has a direct impact on the performance of the adhesive, with polymerization mechanisms occurring at up to 50 wt% of citric acid. Over 50 wt%, the fraction of unreacted citric acid increases, which has a negative impact on the adhesive capacity of the bio-adhesive, by reducing the wood/adhesive interactions. Additionally, it was also observed that the temperature of curing is a determinant in the promotion of chemical phenomena associated with the bonding capacity of the bio-adhesives, and also has an undesired impact on antioxidant properties, by reducing them at higher temperatures.

Particleboards were produced with a target density of 700 kg m^−3^, and the impact of the thickness and pressing temperature on their physical-mechanical performance and antioxidant properties was evaluated. The results obtained corroborate the positive effect of citric acid up to 50 wt% on the mechanical resistance of the panels, reaching an internal bond strength of 0.41 MPa and a bending strength of 7.73 MPa for panels with a 4 mm thickness. Although increasing the thickness to 8 mm led to a substantial reduction in mechanical performance, the increase in pressing temperature up to 220 °C allowed a particleboard that fulfills the requirements of PB Type P1 according to EN 312:2010 to be obtained.

The antioxidant activity of composite panels was attributed to the antioxidant capacity of polyphenolic extract, with a significant impact of pressing temperature on this property. By pressing at 220 °C, the composite panel presents c.a. 45% inhibition of oxidation, which evidences its great potential to be used in active packaging solutions.

In conclusion, the use of forestry by-products from wood packaging to produce novel and sustainable solutions for packaging in the food industry, with improved properties in terms of antioxidant capacity, may provide an excellent opportunity not only to provide added-value to non-useful residues, but also to contribute to an environmentally friendly economy.

## Figures and Tables

**Table 1:** Physical-mechanical properties of PBCA_*X* composite panels (4 mm of thickness) produced through hot-pressing at 160 °C for 10 min.

Reference	Density (kg m^−3^) ^1^	Moisture Content, MC (%) ^1^	Internal Bond Strength, IB (MPa) ^1^	Bending Strength, BS (MPa) ^1^	Modulus of Elasticity, MOE (MPa) ^1^	Thickness Swelling 1 h, TS (%)
PBCA_0	699 ± 31 ^a^	3.7 ± 0.1 ^a^	0.25 ± 0.01 ^a^	5.75 ± 1.37 ^a^	1620 ± 339 ^a^	i.d. ^a^
PBCA_25	682 ± 28 ^a^	5.1 ± 0.1 ^b^	0.31 ± 0.03 ^b^	8.05 ± 0.98 ^b^	1715 ± 21 ^a^	132 ± 5 ^b^
PBCA_50	721 ± 40 ^a^	4.5 ± 0.2 ^c^	0.41 ± 0.01 ^c^	7.73 ± 0.49 ^b^	1750 ± 339 ^a^	102 ± 10 ^c^
PBCA_75	723 ± 18 ^a^	5.0 ± 0.1 ^b^	0.32 ± 0.02 ^b^	9.92 ± 0.30 ^c^	1950 ± 212 ^a^	85 ± 13 ^d^
PBCA_100	708 ± 41 ^a^	5.4 ± 0.1 ^b^	0.33 ± 0.04 ^b^	8.21 ± 0.33 ^b^	1645 ± 21 ^a^	89 ± 8 ^d^
PB Type P1 *	n.r.	n.r.	0.31	11.5	n.r.	n.r.

^1^ Values are presented as mean ± SD (*n* = 3); i.d.—impossible to determine. n.r.—no requirements; * according to EN 312:2010. Values in the same column superscripted by different letters are significantly different at *p* < 0.05.

**Table 2:** Physical-mechanical properties of PBCA_*X* composite panels (8 mm of thickness) produced through hot-pressing at 160 °C for 10 min.

Reference	Density (kg m^−3^) ^1^	Moisture Content, MC (%) ^1^	Internal Bond Strength, IB (MPa) ^1^	Bending Strength, BS (MPa) ^1^	Modulus of Elasticity, MOE (MPa) ^1^	Thickness Swelling 1 h, TS (%)
PBCA_0	703 ± 6 ^a^	4.3 ± 0.3 ^a^	0.07 ± 0.01 ^a^	8.94 ± 1.28 ^a^	2305 ± 841 ^a^	i.d. ^a^
PBCA_25	703 ± 12 ^a^	4.4 ± 0.1 ^a^	0.14 ± 0.03 ^b^	9.27 ± 1.12 ^a^	1905 ± 304 ^a^	175 ± 13 ^b^
PBCA_50	702 ± 5 ^a^	4.8 ± 0.1 ^b^	0.19 ± 0.03 ^c^	8.56 ± 0.76 ^a^	1925 ± 21 ^a^	142 ± 5 ^c^
PBCA_75	690 ± 7 ^a^	5.1 ± 0.1 ^b^	0.07 ± 0.02 ^a^	5.59 ± 0.31 ^b^	1395 ± 7 ^b^	138 ± 10 ^c^
PBCA_100	698 ± 9 ^a^	5.2 ± 0.1 ^b^	0.05 ± 0.01 ^a^	5.93 ± 0.07 ^b^	1175 ± 49 ^b^	138 ± 2 ^c^
PB Type P1 *	n.r.	n.r.	0.28	10.5	n.r.	n.r.

^1^ Values are presented as mean ± SD (n = 3); i.d.—impossible to determine. n.r.—no requirements; * according to EN 312:2010. Values in the same column superscripted by different letters are significantly different at *p* < 0.05.

**Table 3:** Effect of pressing temperature on the physical-mechanical properties of PBCA_*50* composite panel (8 mm thickness).

Pressing Temperature	Density (kg m^−3^) ^1^	Moisture Content, MC (%) ^1^	Internal Bond Strength, IB (MPa) ^1^	Bending Strength, BS (MPa) ^1^	Modulus of Elasticity, MOE (MPa) ^1^	Thickness Swelling 1 h ^1^, TS (%)
160	702 ± 5 ^a^	4.8 ± 0.1 ^a^	0.19 ± 0.03 ^ac^	8.56 ± 0.76 ^a^	1925 ± 21 ^a^	142 ± 5 ^a^
180	703 ± 3 ^a^	4.9 ± 0.2 ^a^	0.17 ± 0.02 ^a^	9.55 ± 0.23 ^a^	2080 ± 353 ^a^	70 ± 3 ^b^
200	694 ± 3 ^a^	4.8 ± 0.1 ^a^	0.20 ± 0.01 ^a^	9.94 ± 1.30 ^a^	2365 ± 318 ^a^	38 ± 4 ^c^
220	699 ± 3 ^a^	4.7 ± 0.2 ^a^	0.28 ± 0.01 ^b^	11.30 ± 1.14 ^b^	2600 ± 141 ^b^	27 ± 3 ^c^
PB Type P1 *	n.r.	n.r.	0.28	10.5	n.r.	n.r.

^1^ Values are presented as mean ± SD (n = 3); n.r.—no requirements; * according to EN 312:2010. Values in the same column superscripted by different letters are significantly different at *p* < 0.05.

## Data Availability

Data are contained within the article.
